# Survival and Complication Rates of Tooth‐Supported Veneered Zirconia Fixed Partial Dentures: A Systematic Review and Metaanalysis

**DOI:** 10.1155/ijod/8282494

**Published:** 2026-10-30

**Authors:** Arul Kumar Sengottaiyan, Vincent Bennani, Arthi Veerasamy

**Affiliations:** ^1^ Department of Oral Rehabilitation Faculty of Dentistry University of Otago Dunedin New Zealand otago.ac.nz

## Abstract

**Statement of Problem:**

The long‐term survival and complication rates of tooth‐supported veneered zirconia fixed partial dentures (FPDs) remain unclear due to heterogeneous and fragmented clinical evidence. Therefore, a systematic review and metaanalysis were conducted to synthesize the available clinical data.

**Purpose:**

The purpose of this systematic review and metaanalysis was to evaluate the survival rates and the incidence of technical and biological complications associated with tooth‐supported veneered zirconia FPDs.

**Materials and Methods:**

Electronic searches were conducted in MEDLINE/Embase, PubMed, Scopus, Web of Science, and Google Scholar databases up to August 2026 according to preferred reporting Items for systematic reviews and metaanalyses (PRISMA) guidelines. Clinical studies evaluating tooth‐supported veneered zirconia FPDs with a minimum follow‐up of 5 years were included. Randomized clinical trials and prospective or retrospective observational studies reporting survival or complication outcomes were considered. Risk of bias was assessed using the ROBINS‐I tool for cohort studies and the Joanna Briggs Institute (JBI) case series checklist for single‐arm studies. Metaanalysis was performed using R software (ggplot and meta package) with a random‐effects model.

**Results:**

The database search identified 2220 records. After removal of 1815 duplicates, 405 records were screened by title and abstract. A total of 180 full‐text articles were assessed for eligibility, and 17 studies met the inclusion criteria and were included in the quantitative synthesis. The pooled survival rate for veneered zirconia was 92% (95% CI: 90%–94%) at 5 years, 86% (95% CI: 81%–89%) at 7 years, and 83% (95% CI: 71%–91%) at 10 years. Veneered zirconia prostheses demonstrated higher technical complications, particularly veneering porcelain chipping.

**Conclusion:**

Tooth‐supported veneered zirconia fixed dental prostheses demonstrated acceptable survival rates over 5–13 years, with most studies reporting survival above 85%. The most frequent complications were chipping of the veneering porcelain.

**Clinical Implication:**

Understanding the survival and complication patterns of tooth‐supported veneered zirconia FPDs provides valuable insights for clinicians in selecting suitable restorative materials and prosthesis designs.

## 1. Introduction

One of the primary goals of prosthetic dentistry is the replacement of missing teeth to restore mastication, speech, and esthetics [1]. Among the treatment options for partially edentulous patients, including dental implants, removable partial dentures, and fixed partial dentures (FPDs), tooth‐supported FPDs remain a predictable option when the abutment teeth are healthy and appropriately positioned [2]. Metal–ceramic fixed dental prostheses have long been considered the conventional method, demonstrating a long‐term success rate exceeding 90% over a 10‐year period [3, 4]. However, a major limitation of metal–ceramic restorations is their relatively inferior esthetics compared with those of all‐ceramic systems [5, 6]. These esthetic limitations have contributed to the increasing use of all‐ceramic materials in fixed prosthodontics.

Clinical outcomes of prosthetic restorations are commonly reported using the parameters of success, survival, and failure. Restoration success is commonly described as the ability of a dental restoration or prosthesis to function as intended while maintaining structural integrity, esthetics, and patient comfort throughout the observation period [7, 8]. Restoration failure is generally defined as any complication that necessitates the replacement of the prosthesis [8, 9]. In contrast, the survival rate describes restorations that remain in situ at the time of evaluation, regardless of whether minor repairs or complications have occurred [10, 11]. Therefore, survival outcomes typically represent the proportion of restorations still functioning clinically over a defined follow‐up period, even if maintenance procedures are required.

Zirconia, a polycrystalline ceramic composed of zirconium dioxide, offers high fracture resistance, favorable flexural strength (900–1200 MPa), excellent biocompatibility, and a tooth‐like color [12, 13]. With the introduction of CAD/CAM technology, yttria‐stabilized tetragonal zirconia polycrystal (Y‐TZP) have been widely used to produce restorations with improved strength and acceptable esthetics [14, 15]. Clinical studies have reported survival rates of zirconia restorations comparable to those of conventional metal–ceramic systems, particularly for single crowns and short‐span FPDs in short and medium‐term follow‐up periods [16].

However, the clinical performance of veneered zirconia restorations in long‐span prostheses remains a subject of concern. Long‐span FPDs, which replace three or more missing teeth or span four or more units, are subjected to greater flexural stress, uneven load distribution, and increased framework deformation under occlusal forces [17]. Although zirconia demonstrates high flexural strength, increasing the span length may increase the risk of mechanical complications, particularly when connector dimensions are inadequate [18]. The additional torque and leverage associated with long‐span FPDs may also increase the risk of complications such as secondary caries, loss of vitality of abutment teeth, debonding of restorations [19, 20], and chipping of the veneering porcelain on zirconia frameworks [19, 21, 22].

Several additional factors may influence the long‐term survival of FPDs, including the type of luting agent, marginal fit, periodontal health of abutment teeth, occlusal scheme, and the presence of parafunctional habits [19]. These biomechanical and clinical considerations highlight the importance of careful patient selection and strict adherence to clinical and laboratory protocols in order to improve prosthesis longevity.

A recent systematic review evaluated the clinical performance of zirconia‐based tooth‐supported fixed dental prostheses across different restoration designs and follow‐up periods [20]. However, the present review specifically focuses on the long‐term clinical performance of veneered zirconia tooth‐supported fixed dental prostheses by restricting inclusion to studies with a minimum follow‐up of 5 years. This approach enables a more comprehensive assessment of restoration longevity, long‐term survival, and late technical and biological complications that may not become apparent during shorter follow‐up periods. Furthermore, the present review provides a focused synthesis of treatment‐related factors reported in long‐term clinical studies, including prosthesis design, connector dimensions, veneering ceramic thickness, and cementation protocols, thereby offering clinically relevant information for the long‐term management of veneered zirconia‐fixed dental prostheses.

Therefore, a systematic evaluation of the available clinical evidence is required to assess the long‐term performance of tooth‐supported veneered zirconia FPDs. Such an analysis may help determine survival rates, identify common complications, and evaluate the risk factors associated with prosthesis failure. A comprehensive assessment may also assist clinicians in making evidence‐based decisions regarding treatment planning, material selection, and clinical protocols.

The purpose of this systematic review was to assess the survival rates of tooth‐supported veneered zirconia FPDs and to analyze the common complications and failure modes associated with these restorations. The primary outcome of this systematic review was the survival rate of tooth‐supported veneered zirconia FPDs. Secondary outcomes included technical complications (such as veneering ceramic chipping, framework fracture, marginal discrepancies, and debonding), biological complications (secondary caries, endodontic complications, and tooth fracture), and factors influencing prosthesis longevity.

## 2. Materials and Methods

This systematic review was conducted in accordance with the preferred reporting Items for systematic reviews and metaanalyses (PRISMA) 2020 guidelines [23]. A protocol was developed prior to the initiation of the study and registered in the Prospective Register of systematic reviews (PROSPERO) (CRD420251130971). The research question was formulated using the PICO framework (Table 1): “What are the survival and complication rates of tooth‐supported veneered zirconia fixed dental prostheses?”

**Table 1:** Population, intervention or exposure, and outcome search strategy (MeSH TERMS).

S. number	Search strategy	
1.	Population	Partially edentulous patients restored with tooth‐supported FPDs
2.	Intervention/Exposure	Tooth‐supported veneered zirconia‐fixed dental prostheses
3.	Comparison	Not applicable
4.	Outcome	Survival rates and incidence of technical and biological complications.

An electronic literature search was conducted using Medline/Embase, PubMed, Scopus, Web of Science, and Google Scholar databases up to August 2026 according to standard database search protocols. The reference lists of all included studies were also manually screened to identify additional relevant publications. The search strategy included combinations of MeSH terms and free‐text keywords, “Fixed dental prosthesis ^∗^” OR fdp ^∗^ OR “denture partial fixed” OR “tooth supported” and zirconia OR zirconium and survival OR complication ^∗^ OR failure OR effective ^∗^ OR outcome ^∗^ OR success ^∗^ OR longevity OR retrospective OR “follow up ^∗^” OR “case control” not “resin bonded” OR cantilever ^∗^ OR “dental veneer ^∗^” OR implant ^∗^ dental prosthesis/or dental abutments/or dental prosthesis design/or denture, partial, fixed/OR “fixed dental prostheses ^∗^” or “denture partial fixed” or FDP ^∗^ or “tooth supported” AND Veneer or Zirconium or Zirconium AND survival or complication ^∗^ or failure ^∗^ or outcome ^∗^ or success ^∗^ or longevity or retrospective or “follow up” or “case control.”

Eligibility criteria were established before study selection (Table 2). Clinical studies evaluating tooth‐supported veneered zirconia‐fixed dental prostheses with a minimum follow‐up of 5 years were included. Both prospective and retrospective clinical studies reporting survival rates, success rates, or clinical complications were considered eligible. Studies involving implant‐supported prostheses, resin‐bonded bridges, partial coverage prostheses, cantilever restorations, case reports, in vitro studies, review articles, and studies with insufficient clinical outcome data were excluded. Only studies published in English were considered (Table 2).

**Table 2:** Inclusion and exclusion criteria.

Inclusion criteria	Exclusion criteria
Articles written in English.	Non‐English literature.
Partially edentulous.	Review articles.
Articles on two or more units of complete coverage FPDs.	Studies that involve materials other than veneered zirconia, including monolithic zirconia.
Minimum 5‐year follow‐up.	In vitro studies, preclinical studies.
In vivo studies.	Studies on removable partial dentures and partial coverage prostheses.
FPDs using veneered zirconia frameworks and that have an antagonist either natural dentition or FPDs.	Single crown, resin‐bonded, and implant‐supported FPD.
Only natural teeth, whether vital or nonvital, are used as abutments.	Animal trials.
Include FPDs for both the anterior and posterior regions, whether in the maxilla or the mandible.	Case reports, case series with less than 10 patients, poster abstracts, interviews, or protocols.
Articles containing information on survival rates, success rates, biological, and mechanical complications.	Data extracted based on charts (no clinical examination of patients) (full crown restorations).
Study design: RCTs, prospective and retrospective observational studies.	Studies with clinically and scientifically not approved experimental protocols (e.g., partial veneer/unusual bonding procedures).
	Studies with no or insufficient information on survival and complication rates, data extraction not possible.
	Restorations on deciduous teeth.

Two independent reviewers (Arul Kumar Sengottaiyan and Vincent Bennani) screened titles and abstracts of all retrieved records. After the removal of duplicates and clearly irrelevant studies, full‐text articles were assessed independently by the same reviewers. Disagreements between reviewers were resolved through discussion with a third reviewer (Arthi Veerasamy). The interexaminer agreement for study selection was assessed using Cohen’s kappa coefficient, which showed strong agreement (*κ* = 0.84). Blinding to authors, institutions, or journal titles was not performed during the screening process.

Data extraction was performed using a predesigned standardized data collection form to ensure consistency. Three reviewers (Arul Kumar Sengottaiyan, Vincent Bennani, and Arthi Veerasamy) extracted the data independently. Extracted information included study characteristics, study design, sample size, follow‐up duration, type of prosthesis, and clinical outcomes, including survival rate, chipping, debonding, endodontic complications, tooth fracture, and periodontal complications (Tables 3, 4, and 5). When reported in the included studies, clinical outcomes were evaluated according to standardized criteria such as the United States Public Health Service (USPHS) or the California Dental Association (CDA) evaluation systems.

**Table 3:** Characteristics of the included studies.

Author year	Setting	Exclusion criteria	Number of FPD	Ant/Post number of units and abutments	Preparation design	Connectors surface area mm^2^	Fabrication method	Surface treatment	Luting agent	Veneered zirconia Fw	Total number of survived units		Total number of failed units	
											*N*	%	*N*	%
Molin and Karlsson (2008) [24]	PC PR & GP University	Bruxism periodontal disease	19	NP 19 × 3 units 2 abutments	Finish line—Chamfer (1 mm) Axial—1 mm, Occlusal—1.5 mm. Taper: NP Height: NP	9 (3 × 3)	CO + CAD‐CAM	H_2_O_2_	GIC—0 RGIC—0 ZnP—10 Re—9 NP—0	Veneered zirconia Fw (0.5 mm wall thickness)	19	100	00	00
Schmitter et al. (2012) [25]	P GP University	Bruxism, periodontal disease	30	11ant/19 post 1 × 4 units, 2 abutments 19 × 5 units 2 and 3 abutments 8 × 6 units 3 abutments 2 × 7 units 3 and 4 abutments	Finish line—Chamfer (0.8 mm) Axial—1 mm, Occlusal—1.5–2 mm. Taper—6° and 8° Height—NP	9 (3 × 3)	CO + CAD‐CAM	Al_2_O_3_	GIC—30 RGIC—0 ZnP—0 Re—0 NP—0	Veneered zirconia Fw (1 mm wall thickness)	22	84.6	4	15.3
Raigrodski et al. (2012) [26]	CC NP University	Bruxism, periodontal disease, Patients with removable	18	0/18 20 × 3 units 2 abutments	Finish line—Rounded shoulder (1 mm) Axial—1–1.5 mm, Occlusal—1.5–2 mm. Taper—10° Height—3 mm	9 (3 × 3)	CO + CAD‐CAM	NP	GIC—0 RGIC—18 ZnP—0 Re—0 NP—0	Veneered zirconia Fw (0.6 mm wall thickness)	17	95	1	5
Kern et al. (2012) [27]	PC NP University	NP	20	0/20 17 × 3 units 3 × 4 units 2 abutments	Finish line—Chamfer (0.8–1 mm) Axial—1 mm, Occlusal—1.5–2 mm. Taper—10°–12° Height—NP	9 (3 × 3)	NP	NP	GIC—0 RGIC—0 ZnP—20 Re—0 NP—0	Veneered zirconia (In‐Ceram Zirconia) Fw (NP)	17	85	3	15
Sorrentino et al. (2012) [28]	PC PR University	Bruxism, periodontal disease	48	0/48 37 × 3 units 2 abutments	Finish line—Rounded chamfer (1 mm) Axial—1.5 mm, Occlusal—1.5–2 mm. Taper—10°–14° Height—≥4 mm	9 (3 × 3)	CO + CAD‐CAM	Ethanol	GIC—0 RGIC—0 ZnP—0 Re—48 NP—0	Veneered zirconia Fw (0.6 mm wall thickness)	48	100	0	0
Burke et al. (2013) [29]	P GP Private Practice	Bruxism, periodontal disease	33	11/22 38 × 3 units 3 × 4 units NP	Finish line‐rounded shoulder (1 mm) Axial—1 mm, Occlusal—2 mm Height—NP	9 (3 × 3)	CO + CAD‐CAM	NP	GIC—0 RGIC—0 ZnP—0 Re—33 NP—0	Veneered zirconia Fw (0.5 mm wall thickness)	32	97	1	3
Monaco et al. (2015) [30]	RC PR University	NP	137	67/70 NP NP	Finish line—Knife edge/chamfer Axial—NP Occlusal—NP Height—NP	NP	CAD‐CAM	NP	GIC—NP RGIC—0 ZnP—NP Re—NP NP—0	Veneered zirconia Fw (NP)	NP	94.70	NP	NP
Lops et al. (2012) [31]	P NP University	NP	24	18/6 24 × 3 units 2 abutments	NP	NP	CAD‐CAM	NP	GIC—0 RGIC—0 ZnP—0 Re—24 NP—0	Veneered zirconia Fw	21	88.9	NP	NP
Rinke et al. (2013) [32]	p 97‐GP 2‐Students University	Bruxism, periodontal disease	99	NP 81 × 3 units 18 × 4 units NP	Finish line—Chamfer (0.8 mm) Axial—1.5 mm, Occlusal—1.5–2 mm. Taper—6°–8° Height—NP	9 (3 × 3)	CO + CAD‐CAM	Al_2_O_3_	GIC—0 RGIC—0 ZnP—99 Re—0 NP—0	Veneered zirconia Fw (0.4 mm wall thickness)	80	83.4	19	3.03
Sola‐Ruiz et al. (2015) [33]	P PR University	Bruxism,periodontal disease	24	27/0 10 × 3 units 10 × 4 units 2 × 5 units 5 × 6 units NP	Finish line—Chamfer/shoulder (1 mm) Axial—1–1.5 mm, Occlusal—1.5–2 mm. Taper—10° Height—NP	7 (NP)	CO + CAD‐CAM	Al_2_O_3_	GIC—0 RGIC—0 ZnP—0 Re—24 NP—0	Veneered zirconia Fw (0.5 mm wall thickness)	23	88.8	3	5
Sax et al. (2011) [34]	PC NP University	NP	26	NP 20 × 3units 5 × 4 units 1 × 5units NP	NP	9 (3 × 3)	CAD‐CAM	NP	GIC—0 RGIC—0 ZnP—0 Re—26 NP—0	Veneered zirconia Fw	26	67	15	33
Chaar et al. (2015) [35]	P 19‐PR 3‐Students University	Bruxism	65	0/65 65 × 3 units 2	Finish line—Chamfer/rounded shoulder Axial—1 mm, Occlusal—1.5–2 mm. Taper—6°–12° Height—NP	12 (4 × 3)‐ Ant and 16 (4 × 4)‐Post	CO + CAD‐CAM	Al_2_O_3_ C_3_H_8_O	GIC—65 RGIC—0 ZnP—0 Re—0 NP—0	Veneered zirconia Fw (In‐Ceram Zirconia) (1 mm occlusal and 0.7 mm circumferentially)	55	93.6	4	6.4
Rinke et al. (2018) [36]	P 97‐GP 2‐Students University	Bruxism, periodontal disease	99	0/99 81 × 3 units 18 × 4 units NP	Finish line—Chamfer (0.8 mm) Axial—1 mm, Occlusal–1.5–2 mm. Taper—6°–8° Height—NP	9 (3 × 3)	CO + CAD‐CAM	Al_2_O_3_	GIC—0 RGIC—0 ZnP—99 Re—0 NP—0	Veneered zirconia Fw (0.4 mm wall thickness)	75	75	24	24.2
Ioannidis and Bindl (2016) [37]	PC NP University	Bruxism, periodontal disease	57	0/57 57 × 3 units 2	Finish line—Shoulder (0.8–1.0 mm) Axial—0.8–1 mm, Occlusal—1.5 mm. Taper—10°–12° Height—3–4 mm	9 (3 × 3)	CO + CAD‐CAM	Al_2_O_3_ alcohol	GIC—0 RGIC—0 ZnP—0 Re—57 NP—0	Veneered zirconia Fw (0.5 mm wall thickness 0.7 mm occlusally)	54	94.7	3	5.3
Teichmann et al. (2018) [38]	P GP University	Bruxism	20	3/17 14 × 3 units 3 × 4 units 2 × 5 units 1 × 6 units NP	Finish line—Chamfer/rounded shoulder (0.8 mm) Axial—0.8 mm, Occlusal–1.5–2 mm. Taper—6°–10° Height—3–4 mm	6 (3 × 2)‐Ant 9 (3 × 3)‐Post	CO + CAD‐CAM	Al_2_O_3_ ethanol	GIC—0 RGIC—21 ZnP—0 Re—1 NP—0	Veneered zirconia Fw (0.6 mm wall thickness 0.7 mm occlusally)	19	95	1	5
Sola‐Ruiz et al. (2022) [39]	PC PR University	Bruxism, periodontal disease		27/0 10 × 3units 10 × 4units 2 × 5units 5 × 6units NP	Finish line—Chamfer/shoulder (1 mm) Axial—1–1.5 mm, Occlusal—1.5–2 mm. Taper—10° Height—NP	7 (NP)	CO + CAD‐CAM	Al_2_O_3_	GIC—0 RGIC—0 ZnP—0 Re—27 NP—0	Veneered zirconia Fw (0.5 mm wall thickness)	22	81.4	5	18.5
Haff et al. (2014) [40]	RC GP Private Practice	NP	33	19/14 22 × 3units 4 × 4units 6 × 5 units 11 × 6 units NP	NP	9 (3 × 3)	NP	NP	GIC—4 RGIC—0 ZnP—10 Re—22 NP—0	Veneered zirconia	30	94	1	3

Abbreviations: Al_2_O_3_, aluminum oxide; Ant, anterior; C_3_H_8_O, isopropyl alcohol; CAD‐CAM, computer‐aided design and computer‐aided manufacturing; CC, cohort clinical study; CO, conventional method (impression, die model, wax pattern); FPD, fixed partial dentures; Fw, framework; GIC, glass ionomer cement; GP, general practitioner; H_2_O_2_, hydrogen peroxide; LDS, lithium disilicate; Mand, mandible; Max, maxilla; *N*, number of units; NP, not provided; P, prospective cohort; PB, practice‐based cohort study; PC, prospective clinical study; Post, posterior; PR, prosthodontist; RC, retrospective cohort study; Re, resin cement; RGIC, resin‐modified glass ionomer cement; ZnP, zinc phosphate cement.

**Table 4:** Survival rates and complication frequency, type, and location.

S. number	Author year	Mean FU wears	Technical				Esthetic	Biological			Complication rates and types	Survival rate (%)
			Chipping (%)	Fracture (%)	Marginal gap (%)	Debonding (%)	Marginal discoloration (%)	Tooth fracture (%)	Secondary caries (%)	Endo complication (%)		
1.	Molin and Karlsson (2008) [24]	5	0	0	31.5	5	21	0	0	0	**Rates—11/19** Debonding—1/19 Discoloration—4/19 Open margin—6/19 Chipping Minor chipping—NP Major chipping—NP Bridge fracture and location—NIL Tooth fracture—NIL Endo—NP Secondary carries—NIL	100
2.	Schmitter et al. (2012) [25]	5	30.7	7.6	0	4.6	0	3.8	0	3.8	**Rates—14/22** Debonding—4/22 Discoloration—NP Open margin Chipping—9/22 Minor chipping—8/22 Major chipping—1/22 Bridge fracture and location—2/22 Tooth fracture—1/22 Endo—1/22 Secondary caries—NIL	84.6
3.	Raigrodski et al. (2012) [26]	5	16.6	0	0	0	0	5.55	0	0	**Rates—4/18** Debonding—NP Discoloration—NIL Open margin—1/18 Chipping—3/18 Minor chipping—1/18 Major chipping—2/18 Bridge fracture and location—NIL Tooth fracture—1/18 Endo—NIL Secondary caries—NIL	95
4.	Kern et al. (2012) [27]	6.2	15	15	0	0	0	0	0	5	**Rates—4/20** Debonding—NIL Discoloration—NIL Open margin—NIL Chipping—3/20 Minor chipping—3/20 Major chipping—NIL Bridge fracture and location—3/20 Tooth fracture—NIL Endo—1/20 Secondary caries—NIL	90
5.	Sorrentino et al. (2012) [28]	5	6.25	0	0	0	0	0	0	0	**Rates—3/48** Debonding—NIL Discoloration—NIL Open margin—NIL Chipping—3/48 Minor chipping—3/48 Major chipping—NIL Bridge fracture and location—NIL Tooth fracture—NIL Endo—NIL Secondary caries—NIL	100
6.	Burke et al. (2013) [29]	5 ± 3	24.2	0	9	0	3	0	0	6.06	**Rates—14/33** Debonding—NIL Discoloration—1/33 Open margin—3/33 Chipping—8/33 Minor chipping—7/33 Major chipping—1/33 Bridge fracture and location—NIL Tooth fracture—NIL Endo—2/33 Secondary caries—NIL	97
7.	Monaco et al. (2015) [30]	5	9.4	2	2	1	1	0	0	1	**Rates—21/137** Debonding—2/137 Discoloration—2/137 Open margin—3/137 Chipping—13/137 Minor chipping—12/137 Major chipping—1/137 Bridge fracture and location—3/137 Tooth fracture—NP Endo—1/137 Secondary caries—NIL	94.7
8.	Lops et al. (2012) [31]	6.5	4	4	0	8	0	4	0	0	**Rates—4/24** Debonding—2/24 Discoloration—NP Open margin—NP Chipping—1/24 Minor chipping—1/24 Major chipping—NIL Bridge fracture and location—1/24 Tooth fracture—1/24 Endo—1/137 Secondary caries—NIL	88.9
9.	Rinke et al. (2013) [32]	7	23.23	0	0	7.07	0	1.01	1.01	4.04	**Rates—35/99** Debonding—7/99 Discoloration—NP Open margin—NP Chipping—23/99 Minor chipping—19/99 Major chipping—4/99 Bridge fracture and location—NIL Tooth fracture—1/99 Endo—4/99 Secondary caries—1/99	83.4
10.	Sola‐Ruiz et al. (2015) [33]	7	14.8	0	0	7.4	0	0	7.4	3.7	**Rates—9/27** Debonding—2/27 Discoloration—NP Open margin—NP Chipping—4/27 Minor chipping—3/27 Major chipping—1/27 Bridge fracture and location—NIL Tooth fracture—NP Endo—1/27 Secondary caries—2/27	88.8
11.	Sax et al. (2011) [34]	10.7 ± 1.3	32	91.5	90.7	0	0	0	27	4	**Rates—30/57** Debonding—NP Discoloration—NP Open margin—39/57 Chipping—16/57 Minor chipping—16/57 Major chipping—0 Bridge fracture and location—3/57 Tooth fracture—NP Endo—3/57 Secondary caries—11/57	67
12.	Chaar et al. (2015) [35]	9.7	37.7	3.38	0	11.2	0	0	8.7	10.8	**Rates—31/65** Debonding—6/65 Discoloration—NP Open margin—NP Chipping—15/65 Minor chipping—7/65 Major chipping—8/65 Bridge fracture and location—2/65 Tooth fracture—NP Endo—5/65 Secondary caries—5/65	93.6
13.	Rinke et al. (2018) [36]	10 ± 3	32.32	0	00	7.07	0	0	6.06	5.05	**Rates—50/99** Debonding—7/99 Discoloration—NP Open margin—NP Chipping—32/99 Minor chipping—31/99 Major chipping—1/99 Bridge fracture and location—NP Tooth fracture—NP Endo—5/99 Secondary caries—6/99	75
14.	Ioannidis and Bindl (2016) [37]	6.3 ± 1.9	28.0	0	0	0	00	3.5	3.5	5.2	**Rates—28/99** Debonding—7/99 Discoloration—NP Open margin—NP Chipping—16/57 Minor chipping—6/57 Major chipping—10/57 Bridge fracture and location—NP Tooth fracture—2/57 Endo—3/57 Secondary caries—2/57	94.7
15.	Teichmann et al. (2018) [38]	10 ± 2.1	40	0	0	0	0	0	00	10.0	**Rates—10/20** Debonding—NIL Discoloration—NIL Open margin—NIL Chipping—8/20 Minor chipping—7/20 Major chipping—1/20 Bridge fracture and location—NIL Tooth fracture—NP Endo—2/20 Secondary carries—NP	95.8
16.	Sola‐Ruiz et al. (2022) [39]	12	25.95	0	0	7.4	0	0	11.11	—	**Rates—12/27** Debonding—2/27 Discoloration—NP Open margin—NP Chipping—7/27 Minor chipping—6/27 Major chipping—1/27 Bridge fracture and location—NIL Tooth fracture—NP Endo—NP Secondary caries—3/27	81.45
17.	Haff et al. (2014) [40]	9.6 ± 1.6	9.7	0	6.5	3.2	0	3.2	9	82	**Rates—14/33** Debonding—1/33 Discoloration—NIL Open margin—2/33 Chipping—3/33 Minor chipping—2/33 Major chipping—1/33 Bridge fracture and location—NIL Tooth fracture—1/33 Endo—5/33 Secondary caries—3/33	94

*Note*: Bold values indicate the reported overall complication frequency, expressed as the number of restorations with complications divided by the total number of restorations evaluated in each study.

Abbreviations: FU, follow‐up; *N*, number of units; NP, not provided.

**Table 5:** Chipping rate in relation to the thickness of veneering porcelain.

Study (author, year)	Chipping	Thickness (mm)	Chipping area
Molin and Karlsson [24]	0/19	0.5.	NP
Schmitter et al. (2012) [25]	9/22	1.0	Minor chipping 8/22 Major chipping 1/22
Raigrodski et al. (2012) [26]	3/18	0.6	Minor chipping 1/18 Major chipping 2/18
Kern et al. (2012) [27]	3/20	NP	Minor chipping 3/20
Sorrentino et al. (2012) [28]	3/48	0.6	Minor chipping 3/48
Burke et al. (2013) [29]	8/33	0.5	Minor chipping 7/33 Major chipping 1/33
Monaco et al. (2015) [30]	13/137	NP	Minor chipping 13/137
Lops et al. (2012) [31]	1/24	NP	Minor chipping 1/24
Rinke et al. (2013) [32]	23/99	0.4	Minor chipping 19/99 Major chipping 4/99
Sola‐Ruiz et al. (2015) [33]	4/27	0.5	Minor chipping 3/27 Major chipping 1/27
Sax et al. (2011) [34]	16/57	NP	Minor chipping 16/57
Chaar et al. (2015) [35]	15/65	1.0	Major chipping 8/65 Minor chipping 7/65
Rinke et al. (2018) [36]	32/99	0.4	Minor chipping 31/99 Major chipping 1/99
Ioannidis and Bindl (2016) [37]	16/57	0.7	Minor chipping 6/57 Major chipping 10/57
Teichmann et al. (2018) [38]	8/20	0.6	Minor chipping 7/20 Major chipping 1/20
Sola‐Ruiz et al. (2022) [39]	7/27	0.5	Minor chipping 6/20 Major chipping 1/20
Haff et al. (2014) [40]	3/33	NP	Minor chipping 2/33 Major chipping 1/33

Abbreviation: NP, not provided.

The risk of bias assessment was performed according to the study design. For nonrandomized cohort studies (prospective, retrospective, or practice‐based), the ROBINS‐I tool was used to assess bias related to confounding factors, participant selection, deviations from intended interventions, missing data, outcome measurement, and selective reporting. For single‐arm case series or small prospective studies without comparison groups, the Joanna Briggs Institute (JBI) critical appraisal checklist for case series was applied (Table 6) [41].

**Table 6:** Risk‐of‐bias assessment for single‐arm/small prospective studies (JBI case series checklist).

Study (author, year)	Study type	Clear inclusion criteria	Standard measurement of condition	Consecutive inclusion of participants	Complete inclusion of participants	Reporting of demographics	Clinical information clearly reported	Outcomes clearly reported	Appropriate statistical analysis	Overall JBI appraisal
Molin and Karlsson (2008) [24]	Prospective clinical	Yes	Yes	Yes	Yes	Yes	Yes	Yes	Yes	Low risk
Kern et al. (2012) [27]	Prospective clinical	Yes	Yes	Unclear	Yes	Yes	Yes	Partial	Yes	Moderate risk
Sorrentino et al. (2012) [28]	Prospective clinical	Yes	Yes	Yes	Yes	Yes	Yes	Yes	Yes	Low risk
Sax et al. (2011) [34]	Prospective clinical	Yes	Yes	Unclear	Yes	Yes	Partial	Partial	Partial	Moderate risk
Ioannidis and Bindl (2016) [37]	Prospective clinical	Yes	Yes	Yes	Yes	Yes	Yes	Yes	Yes	Low risk
Sola‐Ruiz et al. (2022) [39]	Prospective clinical	Yes	Yes	Yes	Yes	Yes	Yes	Yes	Yes	Low risk
Raigrodski et al. (2012) [26]	Case series	Yes	Yes	Unclear	Yes	Yes	Partial	Partial	Partial	Moderate risk

The certainty of evidence was assessed using the Grading of Recommendations Assessment, Development, and Evaluation (GRADE) approach for the primary outcome (survival rate) and the secondary outcome (veneering porcelain chipping). As the review primarily included prospective and retrospective observational studies, the certainty of evidence initially started low.

Metaanalysis was performed using R software (ggplot and meta package) with a random‐effects model. Survival rates at 5, 7, and 10 years were extracted directly from the included studies or estimated from Kaplan–Meier survival curves, when necessary. The inverse‐variance method with logit transformation of proportions was used to stabilize variance and calculate pooled estimates for complication rates and survival outcomes. Because of expected clinical and methodological heterogeneity among studies, a random‐effects model (the DerSimonian–Laird estimator for *τ* ^2^) was used.

Subgroup analysis was conducted for 7 prospective cohort studies [25, 31–33, 35, 36, 38], 2 retrospective cohort studies [30, 40], and a prospective cohort study conducted in a practice‐based clinical setting [29]. However, due to the limited number of studies and variability in follow‐up periods, formal statistical subgroup comparisons were not performed, and the results were reported descriptively.

Statistical heterogeneity among studies was assessed using the Cochran *Q* test, and the magnitude of heterogeneity was quantified using the *I* ^2^ statistic and between‐study variance (*τ* ^2^). A 95% confidence interval (CI) was calculated for all pooled estimates. Funnel plots were visually inspected to assess potential publication bias; however, formal statistical tests were not performed because fewer than 10 studies were available for each pooled outcome.

## 3. Results

The database search yielded a total of 2220 articles, of which 643 were from MEDLINE, 453 from PubMed, 562 from Scopus, 434 from Web of Science, 113 from Google Scholar, and 15 from other sources. After the removal of 1815 duplicate records, 405 articles remained and were screened based on title and abstract. Of these, 221 records were excluded because they were irrelevant to the study objective, 184 reports were sought for retrieval, 4 reports could not be retrieved, and 180 full‐text articles were assessed for eligibility. After applying the inclusion and exclusion criteria (Table 2), 17 studies met the eligibility criteria and were included in the final review. The study selection process is illustrated in the PRISMA flow diagram (Figure 1).

**Figure 1:**
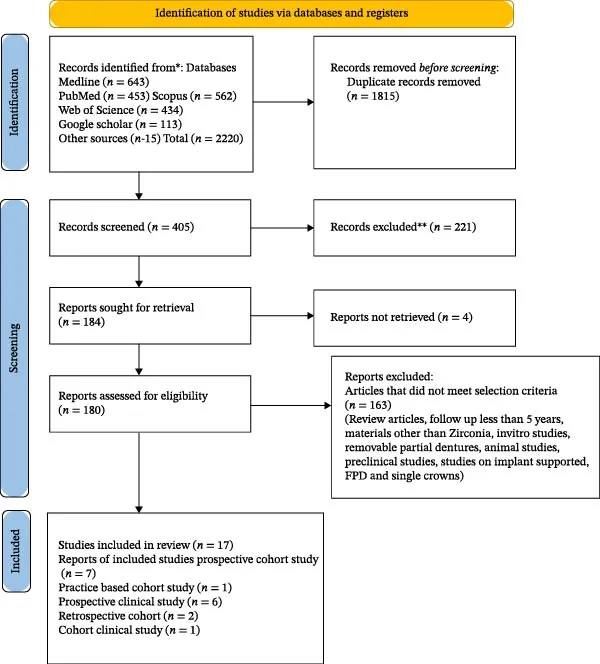
Flow diagram of study selection according to the PRISMA statement.

Risk‐of‐bias assessment using the ROBINS‐I tool indicated that seven studies had a low risk of bias [29, 30, 32, 33, 35, 38, 40], while three studies showed a moderate risk [25, 31, 36] (Figure 2a, b). According to the JBI critical appraisal checklist for case series, four studies demonstrated low risk of bias [24, 28, 37, 39] and three studies showed moderate risk [26, 27, 34]. All 17 studies were included in the quantitative synthesis, and the results of the quality assessment are summarized in Table 6.

**Figure 2:**
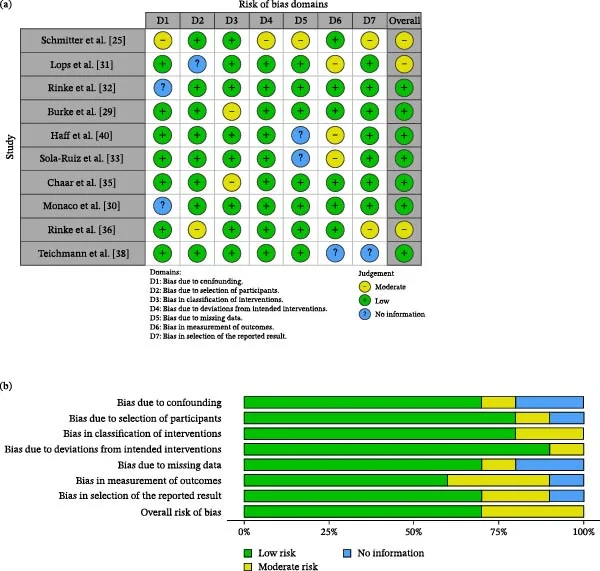
Risk‐of‐bias assessment for cohort studies using the ROBINS‐I tool. (a) Individual studies. (b) Overall studies.

Regarding study design, the included studies comprised 15 prospective studies, including eight prospective cohort studies [25, 29, 31–33, 35, 36, 38]; six prospective clinical studies [24, 27, 28, 34, 37, 39]; one prospective case series [26], and two retrospective cohort studies [30, 40] (Table 3). Follow‐up periods varied across studies. Seven studies reported a 5‐year follow‐up [24–30], three studies reported a 7‐year follow‐up [31–33], five studies reported a 10‐year follow‐up [34–38]. While one study reported a 12‐year follow‐up [39], and one study reported a 13‐year follow‐up [40] (Tables 3 and 4). The number of prostheses evaluated ranged from 18 FPDs [26] to 137 FPDs [30]. Most studies were conducted in university or specialist clinical settings, whereas two studies were performed in a private practice.

With respect to restoration location, most studies evaluated posterior restorations [25, 29, 32, 34–38, 40], whereas three studies focused on anterior restorations [31, 33, 39]. All included studies used CAD–CAM‐fabricated zirconia frameworks. Surface treatment protocols varied, with several studies reporting alumina airborne‐particle abrasion prior to cementation [25, 32, 35–38]. One study reported ethanol cleaning of the intaglio surface before cementation [28]. Various luting agents were used across studies, including glass ionomer cement (GIC), resin cement, zinc phosphate cement, and resin‐modified GIC (RMGIC). The overall follow‐up period ranged from 5 to 13 years, with a mean duration of 7.7 ± 2.71 years.

Most abutment preparations included rounded shoulder or chamfer finish lines, with ~1–2 mm occlusal reduction and about 1 mm axial reduction. Connector dimensions generally ranged between 0.4 and 1 mm in wall thickness, and prosthesis fabrication was predominantly performed using the CAD‐CAM technology.

The reported survival rates of veneered zirconia FPDs ranged from 84.6% to 100% at 5 years, from 83.4% to 88.9% at 7 years, and from 67% to 95.8% at 10 years. The survival rates at 12 and 13 years were 81.45% and 94%, respectively. The highest survival rates were reported by Molin and Karlsson [24] and Sorrentino et al. [28] (100%), whereas the lowest survival rate was reported by Sax et al. [34] (67%) after a mean follow‐up of 10.7 years. Chaar et al. [35] reported a decline in survival from 83.4% at 7 years to 75% at 10 years, suggesting the possibility of late failures. Veneered zirconia FPDs demonstrated favorable long‐term outcomes, with Haff et al. [40] reporting the longest follow‐up period of 13 years with a survival rate of 94%.

Technical, biological, and esthetic complications were evaluated across the included studies. Ceramic chipping was the most frequently reported technical complication, particularly in posterior FPDs, with incidence rates ranging from 4% [31] to 40% [38]. Other reported complications included marginal discrepancies, framework fracture, and debonding. Chipping was classified as minor or major according to previously established criteria [42]. Major chipping was defined as fracture of the veneering ceramic requiring repair, or replacement or affecting prosthesis function [42, 43]. Minor chipping referred to small cohesive fractures that did not compromise restoration function and did not require repair or replacement [42, 43]. Minor chipping was more commonly observed, whereas major chipping occurred less frequently but required clinical intervention. Teichmann et al. [38] reported the highest chipping rate (40%) (Table 5).

Veneering thickness and fabrication technique were identified as important factors influencing chipping rates. Increased chipping was reported in restorations with veneering thickness ≥ 1 mm [25, 35]. In contrast, restorations with thinner veneering layers (≤0.5–0.6 mm) showed lower chipping rates [31, 37, 40]. Processing methods also influenced outcomes; pressed veneering and optimized layering techniques demonstrated lower chipping incidence compared with conventional layering techniques [28, 29] (Tables 3 and 4).

Biological complications were relatively infrequent and included secondary caries, tooth fracture, and endodontic complications. Six studies reported framework fractures [25, 27, 31, 34, 35, 40]. For example, Schmitter et al. [25] reported two framework fractures, whereas Sax et al. [34] reported three fractures that required replacement after 10 years. Rinke et al. [32] observed four framework fractures during a 7‐year follow‐up period (Table 4).

Loss of retention or debonding was reported in 10 studies [24, 30–33, 36, 39, 40]. Rinke et al. [32, 36] reported that four FPDs experienced loss of retention resulting in failure, while seven were successfully managed through recementation. Chaar et al. [35] reported six instances of loss of retention. Marginal discrepancies were reported in five studies [24, 29, 30, 34, 40], with Sax et al. [34] reporting the highest incidence (90.7%). Regarding biological complications, Sax et al. [34] reported secondary caries in 27% of cases, although this did not significantly influence overall survival. Endodontic complications were rarely reported, and tooth fractures were minimal [40] (Tables 3 and 4).

Success rates were generally lower than survival rates because complications such as chipping did not necessarily require prosthesis replacement. For example, Rinke et al. [36] reported a survival rate of 75% but a success rate of 40%, largely due to complications such as chipping and secondary caries. Similarly, Burke et al. [29] reported a 97% survival rate but a 75% success rate, primarily because of veneering ceramic chipping.

Subgroup analysis showed that eight prospective cohort studies [25, 29, 31–33, 35, 36, 38] demonstrated a pooled survival rate of 85.9% (95% CI: 79.0–90.8) with moderate heterogeneity (*I* ^2^ = 56%), whereas two retrospective cohort studies [30, 40] showed a pooled survival rate of ~94.7% (95% CI: 90.4–97.1) with minimal heterogeneity (Table 7; Figures 3 and 4).

**Figure 3:**
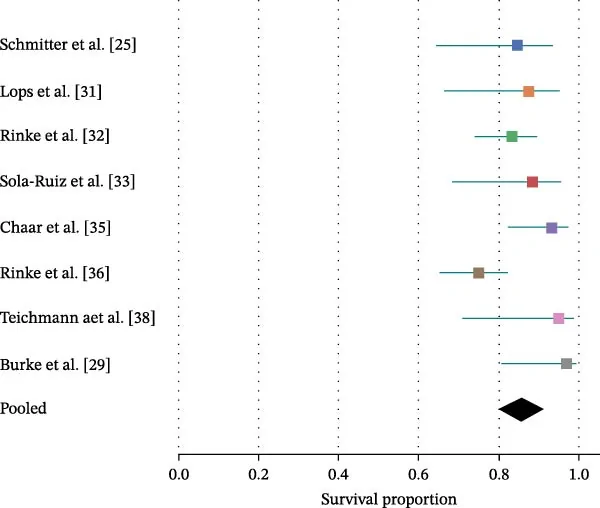
Forest plot–survival rate for prospective cohort studies.

**Figure 4:**
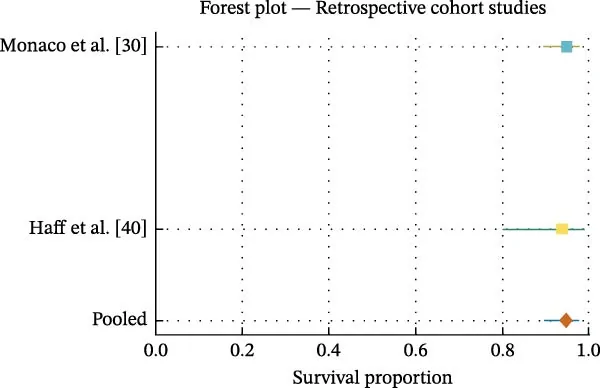
Forest plot–survival rate for retrospective studies.

**Table 7:** Subgroup analysis based on study design.

Study design	Number of studies	Approx. pooled survival (%)	95% CI (%)	*I* ^2^ (%)
Prospective cohort study [25, 29, 31–33, 35, 36, 38]	7	85.9	79.0–90.8	55.9
Retrospective cohort study [30, 40]	2	94.7	90.4–97.1	0

Metaanalysis showed a pooled 5‐year survival rate of 92% (95% CI: 90%–94%), with no statistical heterogeneity (*I* ^2^ = 0%) (Figure 5a, b) (Table 8). When analysis was limited to 9 studies with available raw data, the pooled survival rate was 90% (95% CI: 85%–94%), also demonstrating minimal heterogeneity (*I* ^2^ = 0%) (Figure 6a, b). The 7‐year pooled survival rate across nine studies was 86% (95% CI: 81%–89%), with moderate heterogeneity (*I* ^2^ = 24%). Five studies reported explicit 7‐year survival data, while 3 required estimation from published Kaplan–Meier curves (Figure 7a, b). Five studies reported 10‐year survival outcomes, with a pooled estimate of 83% (95% CI: 71%–91%), although heterogeneity was substantial (*I* ^2^ = 76%) (Figure 8a, b). When calculated using counts‐only data, the survival rate was 86.4% (95% CI: 75.1–93.1) with minimal heterogeneity (*I* ^2^ = 1%). Overall, survival rates tended to decrease with longer follow‐up periods (Figure 9).

**Figure 5:**
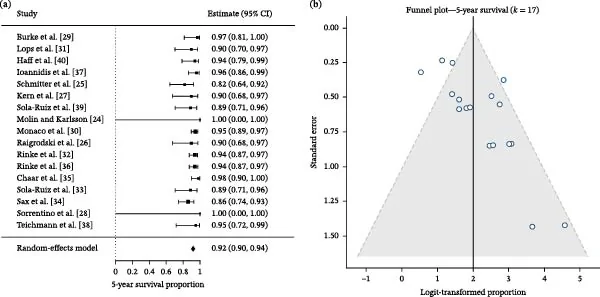
(a) Five‐year pooled survival for 17 studies from approximate values with the Kaplan–Meier curve. (b) Five‐year survival–funnel plot.

**Figure 6:**
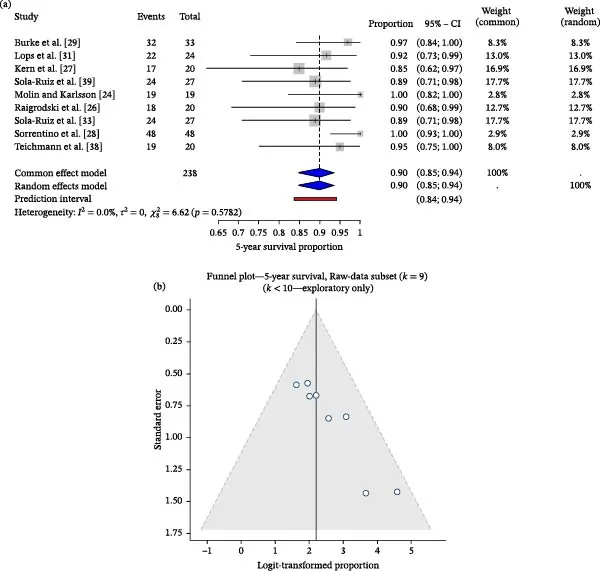
(a) Five‐year pooled survival for 9 studies with available raw data. (b) Five‐year survival for 9 studies with available raw data–funnel plot.

**Figure 7:**
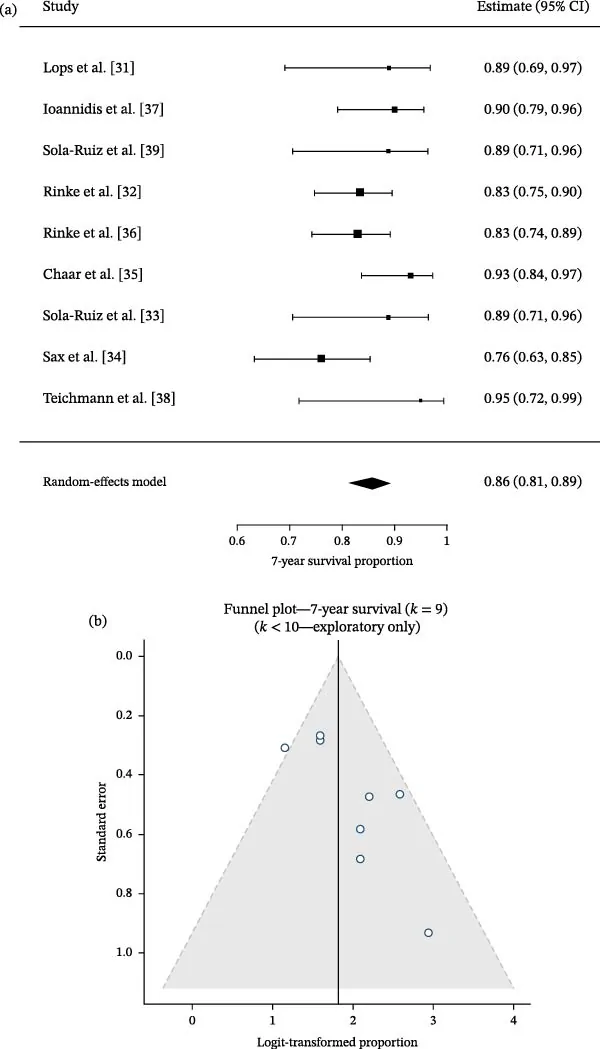
(a) Seven‐year survival rate. (b) Seven‐year survival rate–funnel plot.

**Figure 8:**
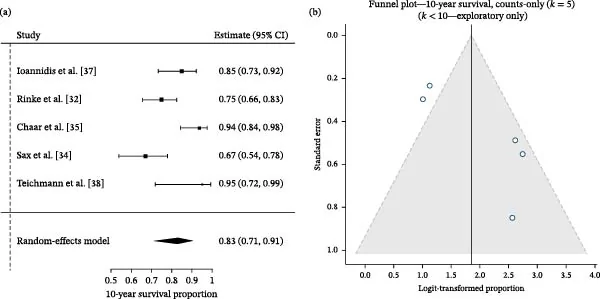
(a) Ten‐year pooled survival for 5 studies from approximate values with the Kaplan–Meier curve. (b) Ten‐year pooled survival for 5 studies–funnel plot.

**Figure 9:**
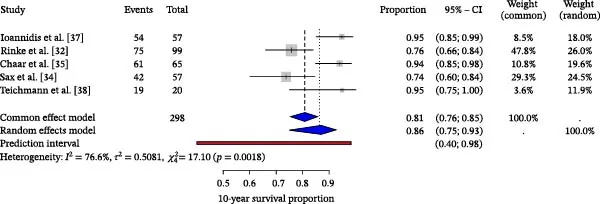
Ten‐year pooled survival rate.

**Table 8:** Pooled survival of zirconia FPDs.

Analysis	*k*	Pooled	95% CI	*I* ^2^ (%)	Tau^2^
5‐year survival (KM, all studies)	17	92.0	89.7–93.9	0	0.00000
5‐year survival (counts‐only)	9	90.0	84.9–93.5	0	0.00000
7‐year survival (KM, all studies)	9	85.7	81.4–89.2	24	0.05365
10‐year survival (KM, all studies)	5	83.0	71.1–90.7	76	0.41679
10‐year survival (counts‐only)	5	86.4	75.1–93.1	1	0.50808

A sensitivity analysis excluding the two studies using In‐Ceram Zirconia frameworks (Chaar et al. [35], Kern et al. [27]) showed no clinically meaningful change in pooled survival or chipping estimates (change ≤ 3 percentage points across all outcomes), indicating that material heterogeneity did not substantially bias the overall pooled results. Heterogeneity at the 10‐year time point remained high after exclusion (*I* ^2^ = 71.8%), suggesting that factors other than framework material composition are the primary contributors to variability at this time point.

Chipping analysis included 17 studies with a total of 815 FPDs (Tables 3 and 5). The pooled 5‐year chipping rate was 20% (95% CI: 16%–26%), with moderate heterogeneity (*I* ^2^ = 61%). The 95% prediction interval ranged from 8% to 42%, indicating variability in chipping incidence across different clinical settings and study designs (Figure 10a, b).

**Figure 10:**
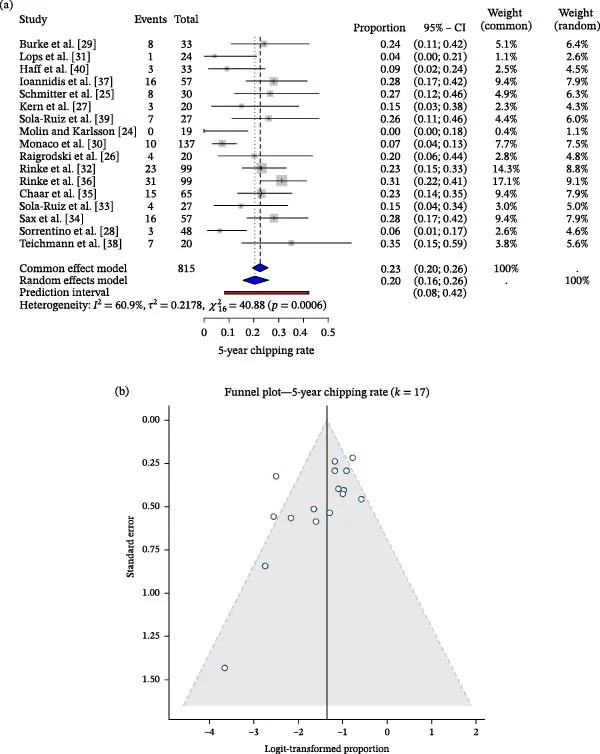
(a) Five‐year chipping rate of the included studies. (b) Five‐year chipping rate–funnel plot.

Funnel plots were constructed for the 5‐year survival and 5‐year chipping rate outcomes to visually assess for asymmetry suggestive of publication bias or small‐study effects; both plots showed a degree of asymmetry, with several studies reporting near‐ceiling proportions (100% survival) positioned asymmetrically relative to the pooled estimate.

The evidence for the 5‐year survival rate was upgraded to moderate certainty because of consistent findings, low statistical heterogeneity, and precise effect estimates. In contrast, the certainty of evidence for the 7‐year and 10‐year survival rates remained low owing to increasing heterogeneity, methodological variability, and differences in clinical protocols across studies. The evidence for veneering porcelain chipping was also rated as low‐certainty because of moderate heterogeneity and variability in outcome assessment.

## 4. Discussion

Based on the findings of the included studies, tooth‐supported veneered zirconia FPDs demonstrated high survival rates with acceptable complication profiles. The metaanalysis estimated pooled long‐term survival and complication rates, demonstrating favorable clinical performance of tooth‐supported veneered zirconia‐fixed dental prostheses. This systematic review examined the outcomes of both prospective and retrospective clinical studies on veneered zirconia‐fixed dental prostheses, with inclusion criteria that were included to summarize the available information regarding survival and success rates, identify common esthetic, biological, and technical complications, evaluate the clinical performance of these reconstructions, and, if possible, provide recommendations for clinical use. The 17 included studies reported follow‐up periods ranging from 5 to 13 years. Overall, FPDs with CAD/CAM‐fabricated veneered zirconia frameworks showed favorable long‐term clinical outcomes, with high survival rates and acceptable success rates over 5–13 years. Nevertheless, chipping of the veneering ceramic remained the most frequently reported complication, affecting the esthetics and reducing restoration success.

Studies conducted in university or specialist clinical settings tended to report slightly better outcomes, likely because of standardized treatment protocols and controlled patient follow‐up. In contrast, studies from private practice settings also demonstrated good survival rates, although variations in tooth preparation, occlusal management, and patient compliance may contribute to differences in complication rates. These observations suggest that the clinical setting and operator expertise can indirectly influence restoration survival.

However, these observations are based on comparisons across different studies and may be influenced by confounding factors, including restoration span, framework design, restoration location, patient selection, follow‐up duration, operator experience, and outcome assessment. Therefore, the influence of clinical setting and operator expertise should be considered as a possible explanatory factor rather than a definitive predictor of clinical outcomes as formal comparative analyses were not performed.

Successful outcomes were commonly associated with rounded chamfer or shoulder finish lines, an axial reduction of at least 1 mm, and adequate connector design. Studies reporting connector dimensions smaller than 9 mm^2^ [34] showed increased fracture and chipping rates, emphasizing the mechanical importance of an appropriate connector size. Furthermore, the included studies consistently indicated that shorter‐span prostheses (3‐unit FPDs) demonstrate higher survival rates and fewer mechanical complications than longer‐span restorations (4–6 units). An increased span length leads to greater flexural stress, reduced rigidity, and an increased risk of framework fracture and veneering ceramic chipping. Consequently, survival tends to decrease as the span length increases.

The overall survival rate across the included studies was 89.4 ± 8.9%, whereas the mean success rate was 81.1 ± 16.2%, with an average observation period of 7.7 ± 2.9 years. The change in survival rates may be due to factors such as the sample size, follow‐up period, patient selection, preparation design, veneering technique, luting protocols, and study design. Studies excluding patients with bruxism or poor oral hygiene generally reported better survival outcomes [25, 35]. These findings highlight the importance of careful patient selection and risk assessment [30]. Long‐span and posterior restorations observed over extended follow‐up periods showed slightly increased material fatigue and a higher incidence of veneering chipping. Despite these complications, survival outcomes were comparable with those reported for conventional metal–ceramic FPDs and single crowns [4, 6]. Connector design and cementation protocols also influenced the clinical performance. Connector dimensions greater than 9 mm^2^ were associated with fewer catastrophic failures [35, 38]. In addition, resin‐based cements demonstrated lower debonding rates compared with zinc phosphate [32, 36] or GICs [35], particularly in posterior and long‐span restorations.

The observed success rates were lower than survival rates because minor complications did not necessarily require the replacement of the prosthesis. Veneering ceramic chipping represented the most frequent technical complication, whereas framework fracture and secondary caries were reported less often. Major chipping events were associated with thicker veneering layers exceeding 0.7 mm, whereas veneering thicknesses between 0.5 and 0.6 mm were associated with fewer failures. Posterior FPDs generally exhibited a higher frequency of complications compared with anterior restorations. Biological complications are relatively uncommon but were evaluated in this review because of their clinical significance. Endodontic complications were rarely observed in vital abutment teeth and were likely related to excessive tooth preparation or preexisting stress conditions. Secondary caries was reported in several studies, highlighting the importance of accurate marginal adaptation and adequate patient oral hygiene. Overall, veneering ceramic chipping was the most common technical complication, affecting ~20% of FPDs within 5 years. The moderate heterogeneity observed for chipping (*I* ^2^ = 61%) may reflect the differences in framework design, veneering techniques, and occlusal loading conditions.

Veneered zirconia restorations generally demonstrated favorable esthetic outcomes, and patient satisfaction was reported to be high in most studies. The longest follow‐up period included in the present review was 13 years, with high survival rates reported by Haff et al. [40]. These findings suggest that veneered zirconia frameworks can provide predictable long‐term clinical performance when appropriate case selection and fabrication protocols are followed. However, veneering ceramic chipping remains a common complication, particularly in posterior load‐bearing regions. To overcome this limitation, monolithic zirconia restorations have been proposed as an alternative approach because they eliminate the veneering layer. Nevertheless, veneered zirconia restorations may still be preferred in anterior regions due to their superior esthetic properties compared with those of monolithic zirconia.

The metaanalysis of the 17 included clinical studies demonstrated pooled survival rates of 92% at 5 years, 86% at 7 years, and 83% at 10 years (Table 8). These findings indicate that the long‐term clinical performance of veneered zirconia FPDs is comparable with that of conventional metal–ceramic restorations while offering improved esthetic potential [3, 4]. The absence of significant statistical heterogeneity for survival outcomes suggests relatively consistent clinical performance across different study populations, treatment settings, and study designs.

The pooled 10‐year survival estimate demonstrated substantial heterogeneity (*I* ^2^ = 76.6%), indicating considerable variability among the included studies (Table 8). This heterogeneity may reflect differences in framework materials, restoration design, connector dimensions, veneering techniques, cementation protocols, patient characteristics, operator experience, and follow‐up methods. Therefore, the 10‐year pooled estimate should be interpreted with caution.

A difference was observed between the Kaplan–Meier‐based survival estimate (83%) and the counts‐only estimate (86.4%). This variation is expected because Kaplan–Meier analysis accounts for censored observations and time‐to‐event data, whereas counts‐only analysis is based on the proportion of surviving restorations at the final follow‐up. These findings should therefore be interpreted as complementary rather than contradictory.

The pooled survival estimate for the retrospective subgroup should be interpreted cautiously because it was based on only two studies. The limited number of studies and potential methodological differences reduce the reliability and generalisability of this subgroup estimate.

The review demonstrated a gradual reduction in survival with increasing follow‐up duration, highlighting the importance of careful patient selection and regular long‐term clinical monitoring. All studies included in the present review evaluated veneered zirconia‐fixed dental prostheses, as monolithic zirconia‐fixed dental prostheses were excluded according to the predefined eligibility criteria. Therefore, the findings of this review are limited to veneered zirconia restorations and cannot be directly extrapolated to monolithic zirconia prostheses. Given the growing clinical use of monolithic zirconia to reduce veneer‐related complications, future well‐designed clinical trials with standardized outcome reporting and longer observation periods are needed to evaluate their long‐term performance and potential advantages.

The included studies evaluated different zirconia framework materials. While most studies investigated CAD/CAM‐fabricated Y‐TZP frameworks, Chaar et al. [35] and Kern et al. [27] evaluated In‐Ceram Zirconia, a glass‐infiltrated zirconia‐reinforced alumina ceramic with different material properties. This material heterogeneity should be considered when interpreting the pooled findings, as the present review did not perform subgroup or sensitivity analyses according to the framework material.

The main strength of this metaanalysis lies in the synthesis of prospective and retrospective clinical data from multiple centers. Nevertheless, several limitations should be considered. These include the limited number of long‐term studies, relatively small sample sizes, and variability in outcome definitions, such as success and survival. In addition, language restrictions may have limited the inclusion of the relevant studies. Some of the included studies lacked randomization and blinding, which may introduce a moderate risk of bias. Furthermore, a formal statistical assessment of small‐study effects/publication bias was not performed because each pooled analysis included fewer than 10 studies, limiting the reliability of tests for funnel plot asymmetry. Funnel plots were therefore inspected descriptively for potential asymmetry, with the findings interpreted cautiously. Another limitation is the inclusion of different zirconia framework materials, including In‐Ceram Zirconia and Y‐TZP, which may have introduced material heterogeneity into the pooled analysis. Overall, the findings indicate favorable long‐term clinical performance of veneered zirconia FPDs, although the certainty of evidence is limited by the observational nature of the included studies.

Zirconia FPDs with CAD/CAM‐fabricated zirconia frameworks appear to be clinically reliable when appropriate clinical and laboratory protocols are followed. Optimal outcomes are achieved when adequate tooth preparation dimensions are maintained, the connector design is properly optimized, and veneering porcelain thickness is limited to ≤0.6 mm in order to reduce chipping risk. In addition, long‐term success requires the careful exclusion of patients with bruxism and other parafunctional habits. The use of CAD‐CAM fabrication techniques may further enhance restoration precision and material integrity compared with conventional methods. Overall, the findings indicate favorable clinical performance of veneered zirconia‐fixed dental prostheses, particularly over the mid‐ to long‐term follow‐up, although the certainty of evidence is limited by the predominantly observational nature of the included studies.

## 5. Conclusions

1. Tooth‐supported veneered zirconia FPDs demonstrated favorable long‐term survival rates over 5–13 years, with most studies reporting a survival above 85%.
2. Veneering porcelain chipping was the most frequent technical complication, with an estimated incidence of ~20% within 5 years, although most events were minor and did not require prosthesis replacement.
3. Survival rates tended to decrease with longer follow‐up periods, suggesting the importance of careful patient selection and long‐term clinical monitoring.

## Author Contributions

**Arul Kumar Sengottaiyan**: conceptualization, developed the research question and study design; methodology, designed the review methodology; investigation, conducted the literature search, study selection, and data extraction; data curation, managed and organized the extracted data; formal analysis, performed the statistical analyses and data synthesis; software, conducted the metaanalysis using appropriate software; validation, verified the accuracy of the extracted data and analyses; visualization, prepared the figures and tables; project administration, coordinated the research activities; resources, acquired the study materials and supporting resources; writing – original draft, prepared the initial manuscript; writing – review and editing, revised the manuscript in response to coauthor feedback. **Vincent Bennani**: conceptualization, contributed to the study design and research objectives; methodology, provided methodological guidance; supervision, supervised the research throughout the project; resources, provided institutional and research resources; funding acquisition, secured funding for the project; validation, critically evaluated the methodology and findings; writing – review and editing, critically reviewed and revised the manuscript. **Arthi Veerasamy**: methodology, contributed to the methodological framework; formal analysis, provided guidance on data interpretation; project administration, assisted with coordination of the research activities; resources, provided academic and research support; supervision, supervised the research and provided scientific guidance; validation, reviewed and verified the analyses and interpretation; writing – review and editing, critically reviewed and revised the manuscript.

## Funding

Open access publishing facilitated by University of Otago, as part of the Wiley ‐ University of Otago agreement via the Council of Australasian University Librarians.

## Disclosure

All authors have read and approved the final version of the manuscript. The corresponding author had full access to all of the data in this study and takes complete responsibility for the integrity of the data and the accuracy of the data analysis.

## Ethics Statement

Ethics approval and informed consent were not required for this study because it is a systematic review and metaanalysis based exclusively on previously published data and did not involve human participants or identifiable personal information.

## Conflicts of Interest

The authors declare no conflicts of interest.

## Data Availability

The authors confirm that the data supporting the findings of this study are available in the article.
